# Exploring the potential mediating role of systemic antibiotics in the association between early-life lower respiratory tract infections and asthma at age 5 in the CHILD study

**DOI:** 10.3389/falgy.2024.1463867

**Published:** 2025-01-21

**Authors:** Maria V. Medeleanu, Myrtha E. Reyna, Darlene L. Y. Dai, Geoffrey L. Winsor, Fiona S. L. Brinkman, Rahul Verma, Ella Nugent, Nashita Riaz, Elinor Simons, Piushkumar J. Mandhane, Meghan B. Azad, Stuart E. Turvey, Theo J. Moraes, Padmaja Subbarao

**Affiliations:** ^1^ Translational Medicine Program, The Hospital for Sick Children, Toronto, ON, Canada; ^2^ Department of Physiology, Temerty Faculty of Medicine, University of Toronto, Toronto, ON, Canada; ^3^ Epidemiology Division, Dalla Lana School of Public Health, University of Toronto, Toronto, ON, Canada; ^4^ Department of Pediatrics, BC Children’s Hospital, University of British Columbia, Vancouver, BC, Canada; ^5^ Department of Molecular Biology and Biochemistry, Simon Fraser University, Burnaby, BC, Canada; ^6^ Division of Respiratory Medicine, Hospital for Sick Children, University of Toronto, Toronto, ON, Canada; ^7^ Department of Pediatrics and Child Health, Section of Allergy and Immunology, University of Manitoba, Winnipeg, MB, Canada; ^8^ Department of Pediatrics, University of Alberta, Edmonton, AB, Canada; ^9^ Faculty of Medicine & Health Sciences, UCSI University, Kuala Lampur, Malaysia; ^10^ Manitoba Interdisciplinary Lactation Centre (MILC), Children’s Hospital Research Institute of Manitoba, Winnipeg, MB, Canada; ^11^ Department of Microbiology and Immunology, University of British Columbia, Vancouver, BC, Canada; ^12^ Division of Respiratory Medicine, Hospital for Sick Children, Toronto, ON, Canada; ^13^ Department of Pediatrics, Temerty Faculty of Medicine, University of Toronto, Toronto, ON, Canada; ^14^ Department of Medicine, McMaster University, Hamilton, ON, Canada

**Keywords:** preschool asthma, respiratory tract infections, antibiotics, mediation analyses, cohort study, clinical epidemiology

## Abstract

**Objective:**

Lower respiratory tract infections (LRTIs) in early life are one of the strongest risk factors for childhood asthma and are often treated with systemic antibiotics (IV or oral). We aimed to explore the association between early-life LRTIs and systemic antibiotics on asthma development and the potential mediating role of antibiotics in this relationship.

**Methods:**

Data were collected as part of the longitudinal, general Canadian population CHILD Study. LRTIs during the first 18 months of life were identified through parental symptom report at regular study visits. Systemic antibiotic use was defined as at least one dose of oral/intravenous antibiotics between birth and the 18-month visit and were further categorized by indication as either given for a respiratory indication (upper or lower respiratory symptoms) or non-respiratory indication. Asthma was diagnosed by in-study pediatricians at the 5-year study visit. Adjusted logistic regression models and mediation analyses via systemic antibiotics use were performed.

**Results:**

Among 2,073 participants included in our analysis, 72 (4.9%) had asthma age 5, and 609 (29.3%) used systemic antibiotics before the 18-month visit. Among children who had taken antibiotics, 61.6% also had an LRTI in that period compared to 49.7% among children without exposure to systemic antibiotics (*p* < .001). Moderate-severe LRTIs before age 18 months were associated with higher odds of 5-year asthma [aOR 4.12 (95%CI 2.04–7.95) *p* < .001]. Antibiotics taken for respiratory indications were associated with higher odds of asthma at age 5 [aOR 2.36 (95%CI 1.59–3.48) *p* < .001]. Children who received systemic antibiotics for only non-respiratory indications during the first 18 months of life were not associated with increased odds of asthma [aOR 1.08 (95%CI 0.44–2.30) *p* = .851]. Using mediation analysis, 21.7% of the association between LRTI and asthma is estimated to be mediated through use of early-life systemic antibiotics. However, a significant direct effect of moderate-to-severe LRTIs on asthma risk remained in adjusted mediation models (*p* = .014).

**Conclusion:**

Through mediation modeling we estimate that the increased risk of asthma at age 5 that is associated with moderate-severe LRTIs in infancy may be partially mediated by systemic antibiotics taken during the first 18 months of life. This underscores the importance of public health strategies focused on antibiotic stewardship and reducing early life LRTIs to mitigate asthma risk.

## Introduction

Asthma is one of the most common chronic diseases among children in the United States and Canada and is characterized by reversible airway obstruction, airway hyper-responsiveness, and airway inflammation (1–3). Lower respiratory tract infections (LRTIs) in the first years of life are one of the strongest risk factors for childhood asthma (4, 5) with recent meta-analyses estimating that children who experienced a LRTI in infancy are up to three times more likely to develop wheezing in adolescence and that this association persists into adulthood (6). Given that by age two years about 80% of children are exposed to a respiratory virus, it is imperative to disentangle the underlying mechanisms and modifiable risk factors driving these associations (7). Recent interest has pointed to analyzing modifiable factors in early life such as the use of systemic antibiotics in the treatment of LRTI.

Antibiotic use in the first year of life has consistently been reported to be strongly associated with later asthma diagnosis (8–12). Moreover, investigators reported that at a population level in British Columbia, Canada, each 10% increase in antibiotic prescriptions under age 1 was associated with a 24% increase in asthma prevalence between 1 and 4 years of age (11). Other studies suggest that the indication for antibiotic prescription may affect the risk of asthma development, noting that systemic antibiotics prescribed to treat respiratory infections (e.g., amoxicillin, penicillin, cephalosporin, and macrolides) resulted in a greater risk of asthma compared to systemic antibiotics prescribed for non-respiratory indications, such as a urinary tract infection (10). Antibiotics taken for non-respiratory infections have also been associated with asthma development but with lower estimated effects (10, 13).

Despite consistent associations between LRTIs in early life and the development of later asthma, their independent or joint effect with early-life antibiotic use has not been thoroughly studied. Specifically, it is unclear whether early-life systemic antibiotics serve as a mediating variable of the association of early life LRTIs and subsequent childhood asthma. In the present study, we analyze data from the longitudinal multi-center CHILD Study to assess the relationship between symptomatic LRTIs in the first 18 months of life and physician-diagnosed asthma by 5 years of age, and to estimate the mediating role of antibiotics use up to 18 months of age.

## Methods

### Study population

The CHILD Study enrolled pregnant mothers during their second or third trimester from the general Canadian population, with an initial recruitment of 3,624 mothers between 2008 and 2012 (14). After healthy delivery at 35 weeks gestation or later, 3,454 mothers remained eligible. The study, spanning four locations across Canada (Vancouver, Winnipeg and Morden-Winkler, Edmonton, and Toronto) and had inclusion criteria of mother's age being >18 years (>19 years in Vancouver), residence within 50 km of the delivery hospital and had consented to cord blood donation. Exclusion criteria covered major congenital abnormalities, respiratory distress syndrome, multiple births, plans to relocate within a year, *in vitro* fertilization-conceived children, and those not spending more than 80% of their time at their primary listed home.

### Lower respiratory tract infections

Parent/caregiver reported history of colds and respiratory symptoms from birth to 18 months of age were collected at regular study intervals (3-, 6-, 12-, and 18-month study visits). We developed a symptom-based LRTI definition after a thorough review of the literature regarding epidemiological definitions of respiratory infections in children [detailed in (15)]. An LRTI was defined as the presence within the last 3 or 6 months of: (i) a cold and (ii) a fever, and (iii) a cough, chest congestion or trouble breathing (Supplementary Figure 1). LRTI severity was classified as mild, or moderate/severe based on health care utilization. Moderate-severe infections were any LRTI that required an unscheduled doctor visit (moderate), a hospital visit or an emergency department visit (severe). Children could have an LRTI with or without wheeze symptoms, as it was not required for our definition to minimize the risk of identifying children already showing potential signs of propensity to asthma (airway hyperresponsiveness).

Given that clinical diagnosis and/or virological testing for all LRTIs was unavailable, we performed validation of our symptom-based LRTI using a subset of CHILD Study participants (365 families) using Viral Score Cards modified from the URECA Wisconsin Upper Respiratory Symptom Survey for Kids (WURSS-K) and nasal swabs for PCR sequencing of nine viruses (Influenza A, Influenza B, Respiratory syncytial virus, Rhinovirus, Enterovirus, Parainfluenza 1, 2 and 3, Metapneumovirus and Adenovirus) (16). Parents/caregivers were asked to call study investigators and fill out the URECA WURSS-K Viral Score Cards by phone during every period of respiratory illness in the first year of life. Infants then were scored as having no symptoms (score = 0), mild symptoms (score 1–3), moderate symptoms (score = 4), or severe symptoms (score ≥5). Infants who received moderate or severe scores underwent nasal swabs that were subsequently PCR sequenced for nine viruses including Influenza A, Influenza B, Respiratory syncytial virus, Rhinovirus/Enterovirus, Parainfluenza 1,2 and 3, Metapneumovirus and Adenovirus. Symptoms associated with positive viral swabs are detailed in Supplementary Table 1. Symptoms of “runny nose”, “wiping nose once per hour”, “cough in the last three days” and “fever” were most prevalent among positive viral swabs (Supplementary Table 1). We performed a receiver operating characteristic (ROC) analysis to identify the diagnostic performance of our LRTI and URTI definitions in this subset of CHILD participants. Any positive viral swab was assigned as true positive reference.

### LRTI definition validation

In our LRTI definition validation sub-study, 365 participants notified the study team of a “cold”. 70% of these colds were assigned “mild” scores while 13% of kids were assigned “severe” scores. Moderate to severe “colds” occurred in 24% of sub-study who then underwent nasal swabs (*n* = 86). Enterovirus/Rhinovirus (ER) (25.58%) and RSV (16.26%) were the most common agents among the positive swabs. A minority were positive for *S.pneumonia* (11.63%) and 13.95% were negative for the entire panel tested (Supplementary Figure 1). In our AUROC analysis, an LRTI definition that included fever had the highest area under the curve (AUC = 0.70) for a positive swab compared to the LRTI definition without fever (AUC = 0.52) suggesting that including fever significantly improved detection of LRTI cases and this definition was therefore used in all subsequent analyses (Supplementary Figure 2, Table 2).

### Antibiotic medication histories

Antibiotics used between birth and the 18-month visit were obtained from CHILD Study medication questionnaires completed by a parent or caregiver at the 3-,6-,12-,18-month visit. Children were classified as exposed or unexposed to early life systemic antibiotics if at least one oral/intravenous antibiotic course was reported between birth and 18-month visit. Early-life systemic antibiotic use from hereon refers to at least one dose of oral/intravenous antibiotics between birth and the 18-month visit.

Antibiotic use was further classified as used for any respiratory or only non-respiratory indications. Children who received systemic antibiotics for any respiratory indications [upper respiratory tract infections, ear infection (otitis media), sinusitis, sore throat, croup, bronchiolitis, bronchitis, pneumonia, combinations of cold, congestion, cough and fever symptoms, influenza, and respiratory distress at birth] were included in the respiratory indication group. Children who only received antibiotics for non-respiratory indications (eye infections, sepsis, urinary tract infections, skin conditions such as eczema, rash, hives, and impetigo or various fungal infections) were included in the non-respiratory indication group. Children who received a systemic antibiotic for both a respiratory and non-respiratory infection were included in the any respiratory antibiotic group. Similarly, if a child received multiple antibiotics during infancy for both reasons, they were included in the any respiratory antibiotic group.

To control for the possibility that prior exposure to antibiotics may affect the risk of early LRTI, participants who received systemic antibiotics at a visit prior to when they reported their first LRTI were excluded from analyses. Finally, to avoid confounding by reverse causation, children who received systemic antibiotics due to wheeze or asthma symptoms were excluded from analyses.

### Asthma diagnosis and wheezing

Pediatric asthma specialists conducted structured interviews with accompanying parent or guardian at in-person clinic visits to identify symptoms and physical findings consistent with asthma (14). Study physicians answered the question: “In your opinion, does this child have asthma? (Yes/Possible/No)”. Children in the “Possible” category were considered as “No asthma”.

### Covariates

Child ethnicity (Caucasian/non-Caucasian), parity (older siblings yes/no), household annual income ($0–$49,999 CAD, $50,000–$99,999, $100,000–$149,999, >$150,000), maternal and paternal physician-diagnosed asthma (ever/never diagnosed), and any prenatal smoke exposure (yes/no) were self-reported at enrollment through questionnaires during the second or third trimester of pregnancy. Infant sex (female/male), gestational age and weight at birth and mode of delivery (cesarian/vaginal) were obtained from birth records. Information regarding the duration (months) and exclusivity (exclusive or partial/none) of breastfeeding in the first 3, 6 and 12 months were collected through repeated questionnaires within the first two years of life. Information on time spent away from home at 18 months was defined as significant time (>7 h per week, yes/no) spent away from home, and was collected through parent questionnaires. Information on recurrent wheezing was collected through clinical assessments performed at the 1-year and 3-year visits. At the clinical visit, parents were asked if the child had a wheezing noise coming from their chest in the past 12 months (yes/no). Recurrent wheezing was defined as 2 or more episodes of wheeze in the last 12 months reported at the 3-, 6- or 12 month CHILD Health Questionnaires.

### Statistical methods

Descriptive statistics are presented as mean (standard deviation, SD) for continuous measures, and frequency (%) for categorical measures. *P*-values were obtained by *t*-test, Fisher's exact test and chi-square test where appropriate.

Adjusted logistic regression analyses were performed using the R *stats* package “glm” function to estimate the odds ratios (OR) and 95% confidence intervals (CI) for the association of any LRTI in early life, LRTI severity (No, Mild, Moderate-Severe) and systemic antibiotic use (No vs. Yes) with asthma diagnosis at 5 years of age. Each exposure was analyzed separately and in combined models to determine their relationships with 5-year asthma development. All models were adjusted by study site, child sex, ethnicity (Caucasian), breastfeeding status at 3-months, prenatal smoke exposure, mode of delivery, family income, time spent away from home at 12-months, presence of older siblings, and parental history of asthma. To explore the impact of indication, we replicated our logistic regression models in children with antibiotics taken for a respiratory indication (any respiratory antibiotic use vs. no systemic antibiotics) and a second set examining the impact of non-respiratory antibiotics (non-respiratory antibiotic use vs. no systemic antibiotics).

Mediation analyses were performed using the Baron & Kenny approach in the R *mediation* package “mediate” function using a mediator model assessing the direct relationship between LRTI severity and antibiotic use, and an outcome model evaluating the effect of these factors on 5-year asthma. To satisfy the binary exposure required in mediation analysis, moderate-severe infections were compared to a combined reference group of children who reported mild or no LRTIs in the first 18 months. The total, direct, and indirect effects (mediation) were estimated, with adjustments made for potential confounders. Confidence intervals were calculated via bootstrapping (*n* = 1,000 simulations). Mediation analysis was also undertaken after excluding children with antibiotics due to non-respiratory indications.

Finally, a sensitivity analysis using the same set of confounders as the primary analysis was performed in non-wheezing children to investigate if the effects remained consistent in children not predisposed to asthma. R version 4.2.2 was used to perform all analyses.

## Results

Out of all participants in the CHILD Study (*N* = 3,454), 3,301 children had at least one respiratory infection questionnaire completed during the first 18 months of life. Among participants with respiratory infection questionnaires available, 2,073 (62.8%) also had an available history of antibiotic medication between birth and 18-month visit (at least two antibiotic questionnaires competed) along with an asthma diagnosis recorded at the 5-year visit (Supplementary Figure 2). These participants were included in subsequent analyses. Of these participants (*n* = 2,073), 52% were male, 65% were Caucasian, 16.5% had prenatal smoke exposure and 37% had a family history of asthma (Table 1). 1,420 (43.0%) of these participants reported at least one LRTI in the first 18 months (Supplementary Table 3, Supplementary Figure 5). Prevalence of any LRTI or most severe LRTI during the first 18 months of life in the CHLD Study are provided in Supplementary Figure 5.

**Table 1:** Demographic and clinical characteristics of CHILD study participants by history of LRTI or systemic antibiotics taken between birth and 18-month visit. All *p*-value comparisons made to “No LRTI” or “No systemic antibiotics” in the first 18 months group (*n* = 2,073).

	Lower respiratory tract infections (LRTI)				Systemic antibiotics						
	No LRTI	Mild LRTI	Moderate-Severe	*p*	No systemic antibiotics	Any systemic antibiotics	*p*	Non-respiratory indication	*p*	Respiratory indication	*p*
	(*n* = 971)	(*n* = 1,026)	(*n* = 76)		(*n* = 1,464)	(*n* = 609)		(*n* = 132)		(*n* = 477)	
Sex assigned at birth, male (%)	499 (51.4)	572 (55.8)	40 (52.6)	.146	760 (51.9)	351 (57.6)	.**020**	80 (60.6)	.068	271 (56.8)	.070
Caucasian, yes (%)	647 (66.6)	677 (66.0)	47 (61.8)	.690	955 (65.2)	416 (68.3)	.195	85 (64.4)	.922	331 (69.4)	.107
Study Site (%)				.221			.**009**		.807		.**006**
Edmonton	145 (14.9)	176 (17.2)	13 (17.1)		241 (16.5)	93 (15.3)		22 (16.7)		71 (14.9)	
Toronto	251 (25.8)	219 (21.3)	20 (26.3)		340 (23.2)	150 (24.6)		30 (22.7)		120 (25.2)	
Vancouver	222 (22.9)	260 (25.3)	20 (26.3)		381 (26.0)	121 (19.9)		30 (22.7)		91 (19.1)	
Winnipeg	353 (36.4)	371 (36.2)	23 (30.3)		502 (34.3)	245 (40.2)		50 (37.9)		195 (40.9)	
LRTI in the first 18 months	–	–	–	–	728 (49.7)	375 (61.6)	**<**.**001**	60 (45.5)	.396	315 (66.0)	**<**.**001**
Age of the first LRTI	–	11.87 (4.14)	10.15 (4.41)	–	11.99 (4.20)	11.24 (4.10)	.**009**	10.28 (4.30)	.**009**	11.39 (4.06)	.**048**
Total LRTI in the first 18 months				–			.**026**		.286		.**041**
1 LRTI reported	–	652 (77.3)	36 (48.0)		465 (78.2)	224 (69.1)		31 (68.9)		193 (69.2)	
2 LRTI reported	–	168 (19.9)	25 (33.3)		110 (18.5)	83 (25.6)		11 (24.4)		72 (25.8)	
3 LRTI reported	–	19 (2.3)	11 (14.7)		16 (2.7)	14 (4.3)		3 (6.7)		11 (3.9)	
4 LRTI reported	–	4 (0.5)	3 (4.0)		4 (0.7)	3 (0.9)		0 (0.0)		3 (1.1)	
Weight for age z-score at birth [mean (SD)]	0.30 (1.00)	0.32 (0.96)	0.29 (0.82)	.84	0.30 (0.96)	0.33 (1.01)	.483	0.08 (1.05)	.**014**	0.40 (0.99)	.**046**
Weight at 3-year, kg [mean (SD)]	15.12 (1.98)	14.98 (1.74)	15.01 (1.86)	.288	15.01 (1.86)	15.13 (1.89)	.178	14.94 (1.70)	.661	15.19 (1.94)	.076
Height at 3-year, kg [mean (SD)]	96.03 (4.01)	95.81 (3.85)	95.97 (4.08)	.481	95.85 (3.88)	96.07 (4.06)	.269	95.47 (4.11)	.285	96.23 (4.04)	.071
Weight at 5-year, kg [mean (SD)]	19.64 (2.98)	19.41 (2.76)	19.28 (2.34)	.136	19.43 (2.79)	19.72 (2.98)	.**031**	19.42 (2.94)	.996	19.80 (2.98)	.**011**
Height at 5-year, kg [mean (SD)]	110.80 (4.77)	110.68 (4.70)	110.49 (4.59)	.769	110.68 (4.66)	110.87 (4.88)	.388	110.18 (5.15)	.252	111.06 (4.79)	.118
Prenatal smoke exposure, yes (%)	166 (17.1)	161 (15.7)	12 (15.8)	.69	242 (16.5)	97 (15.9)	.785	18 (13.6)	.460	79 (16.6)	1.00
Prenatal annual family income, CAD (%)				.741			.623		.358		.821
$0–$49,999	105 (10.8)	114 (11.1)	12 (15.8)		164 (11.2)	67 (11.0)		14 (10.6)		53 (11.1)	
$50,000–$99,999	305 (31.4)	314 (30.6)	23 (30.3)		468 (32.0)	174 (28.6)		34 (25.8)		140 (29.4)	
$100,000–$149,999	251 (25.8)	276 (26.9)	14 (18.4)		374 (25.5)	167 (27.4)		44 (33.3)		123 (25.8)	
> $150,000	215 (22.1)	230 (22.4)	21 (27.6)		323 (22.1)	143 (23.5)		28 (21.2)		115 (24.1)	
*Prefer not to say*	95 (9.8)	92 (9.0)	6 (7.9)		135 (9.2)	58 (9.5)		12 (9.1)		46 (9.6)	
Time away from home <18 m, yes (%)	434 (55.4)	515 (59.1)	42 (63.6)	.19	654 (55.0)	337 (63.3)	.**001**	60 (56.1)	.911	277 (65.2)	**<**.**001**
Older siblings, yes (%)	369 (38.0)	482 (47.0)	43 (56.6)	**<**.**001**	605 (41.3)	289 (47.5)	.**012**	62 (47.0)	.243	227 (47.6)	.**019**
Delivery mode, cesarian section (%)	233 (24.0)	251 (24.5)	18 (23.7)	.965	343 (23.4)	159 (26.1)	.215	43 (32.6)	.**025**	116 (24.3)	.738
Hospital breastfeeding status				.359			.**038**		.**004**		.312
Exclusive	622 (74.3)	664 (76.4)	51 (79.7)		963 (77.2)	374 (71.5)		71 (63.4)		303 (73.7)	
Partial	197 (23.5)	184 (21.2)	10 (15.6)		256 (20.5)	135 (25.8)		36 (32.1)		99 (24.1)	
None	18 (2.2)	21 (2.4)	3 (4.7)		28 (2.2)	14 (2.7)		5 (4.5)		9 (2.2)	
3-month breastfeeding status				.130			.052		.159		.130
Exclusive	614 (63.2)	627 (61.1)	41 (53.9)		925 (63.2)	357 (58.6)		77 (58.3)		280 (58.7)	
Partial	234 (24.1)	284 (27.7)	21 (27.6)		376 (25.7)	163 (26.8)		33 (25.0)		130 (27.3)	
None	123 (12.7)	115 (11.2)	14 (18.4)		163 (11.1)	89 (14.6)		22 (16.7)		67 (14.0)	
6-month breastfeeding status				.584			.073		.466		.113
Exclusive	178 (18.4)	205 (20.0)	12 (15.8)		292 (20.0)	103 (16.9)		22 (16.8)		81 (17.0)	
Partial	592 (61.2)	603 (58.9)	44 (57.9)		878 (60.2)	361 (59.4)		78 (59.5)		283 (59.3)	
None	197 (20.4)	216 (21.1)	20 (26.3)		289 (19.8))	144 (23.7)		31 (23.7)		113 (23.7)	
Inhalant sensitization at 1-year, yes (%)	35 (3.7)	39 (3.9)	3 (4.1)	.981	55 (3.8)	22 (3.7)	.992	3 (2.3)	.623*	19 (4.1)	.919
Atopy 1 year, yes (%)	120 (12.8)	126 (12.5)	10 (13.5)	.956	188 (13.1)	68 (11.5)	.345	18 (14.1)	.868	50 (10.8)	.208
Atopy 3 year, yes (%)	126 (13.7)	141 (14.3)	10 (14.3)	.918	187 (13.3)	90 (15.7)	.202	21 (16.7)	.364	69 (15.4)	.315
Atopy 5 year, yes (%)	180 (19.4)	193 (19.5)	18 (24.3)	.582	262 (18.7)	129 (21.8)	.121	30 (23.1)	.270	99 (21.5)	.213
Recurrent wheezing 1-year, Yes (%)	54 (5.6)	90 (8.8)	25 (32.9)	**<**.**001**	88 (6.0)	81 (13.3)	**<**.**001**	11 (8.3)	.385	70 (14.7)	**<**.**001**
Recurrent wheezing 3-year, Yes (%)	73 (7.6)	93 (9.2)	17 (22.4)	**<**.**001**	108 (7.5)	75 (12.4)	**<**.**001**	11 (8.4)	.832	64 (13.5)	**<**.**001**
Asthma 3-year, yes (%)	48 (5.1)	56 (5.6)	15 (20.5)	**<**.**001**	66 (4.6)	53 (8.9)	**<**.**001**	6 (4.6)	1.00*	47 (10.2)	**<**.**001**
Asthma 5-year, yes (%)	48 (4.9)	67 (6.5)	14 (18.4)	**<**.**001**	72 (4.9)	57 (9.4)	**<**.**001**	7 (5.3)	.833*	50 (10.5)	**<**.**001**
Parental history of asthma, Yes (%)	356 (36.7)	385 (37.5)	36 (47.4)	.178	538 (36.7)	239 (39.2)	.308	39 (29.5)	.120	200 (41.9)	.**049**

Bold indicates significant *p* values.

* Fisher's exact test was used instead of Chi-square testing for any categorical variables where *n* < 5.

### LRTI and antibiotic Use

1,464 of the 2,073 included participants reported no systemic (oral or IV) antibiotics taken in the first 18 months of life and 609 (29.4%) reported taking 1 or more dose of systemic antibiotics during this period. Of these participants, 132 (6.4%) reported antibiotics taken for only non-respiratory indications and 477 (23.0%) reported taking antibiotics for any respiratory indication (Table 1).

Participants who reported an LRTI in the first 18 months of life were more likely to have older siblings (56.6% of Moderate-severe, 47.0% of Mild vs. 38.0% of No LRTI group), more recurrent wheezing (32.9% Moderate-severe vs. 81.8% Mild vs. 5.6% of the No LRTI group), and more 3-year and 5-year asthma (Table 1). Participants who received systemic antibiotics were significantly more likely to be male (57.6 vs. 51.9%), be from the Toronto or Winnipeg study sites (*p* = .009), significantly more likely to have early and more frequent LRTIs in the first 18 months than children with no systemic antibiotics use (Table 1). These children were also significantly more likely to spend time away from home at 18-months (63.3% vs. 55.0%), have older siblings (47.5% vs. 41.3%), not be exclusively breastfed at hospital discharge, 3- or 6-months (*p* = .038, .052, .073), and had higher rates of 1- and 3-year recurrent wheeze (8.3% vs. 6.0% and 8.4% vs. 6.0%), and 3- and 5-year physician diagnosis of asthma (8.9% vs. 4.6% *p* < .001, 9.4% vs. 4.9%) (Table 1). Compared to children without systemic antibiotics, participants with antibiotics taken for a respiratory indication were significantly more likely to have LRTIs in the first 18 months, had higher weight for age z-score at birth, higher weight at the 5-year visit, spent more time away from home at 18-months, were more likely to have older siblings, had higher rates of 1- and 3-year recurrent wheeze (14.7% vs. 6.0% and 13.5% vs. 7.5%), and 3- and 5-year physician diagnosis of asthma (10.2 and 10.5% vs. 4.6 and 4.9%) (Table 1). Children who received systemic antibiotics for a respiratory indication by the 18-month visit had significantly higher rates of parental history of asthma compared to the no systemic antibiotics group (41.9% vs. 36.7%) (Table 1).

### LRTI and systemic antibiotics in the first 18 months are associated with asthma at 5-years

**Figure 1:**
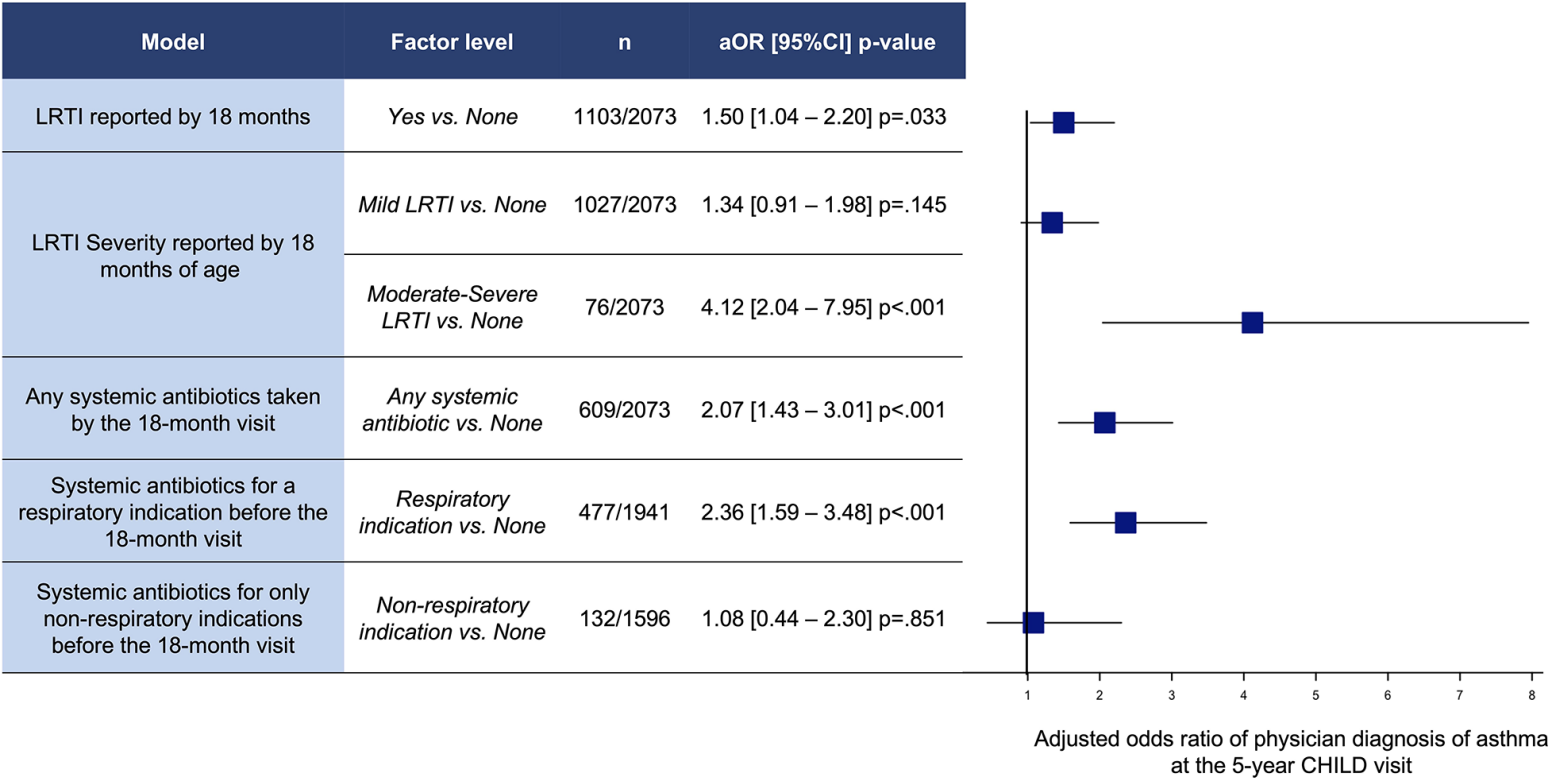
Adjusted odds ratios (OR) and 95% confidence intervals (CI) of physician diagnosis of asthma at 5 years of age for LRTIs and antibiotics between birth and 18-month visit. Models adjusted by site, child sex, Caucasian, breastfeeding <3 m, prenatal smoke exposure, mode of delivery, family income, away from home <12 m, older siblings, and parental history of asthma.

Participants who reported a LRTI in the first 18 months of life had greater odds of 5-year asthma (aOR 1.50 [95%CI 1.04–2.20] *p* = .033 (*n* = 1,103/2,073) (Figure 1). Children with moderate-severe LRTIs had significantly higher odds of 5-year asthma [aOR 4.12 (95%CI 2.04–7.95) *p* < .001] (*n* = 76/2,073) (Figure 1). Children with only mild LRTIs in the first 18 months of life had no significant increase in odds of 5-year asthma compared to the no LRTI group [aOR 1.34 (95%CI 0.91–1.98) *p* = .145] (*n* = 1,026/2,073) (Figure 1).

Systemic antibiotics taken by the 18-month visit was associated with a significantly increased adjusted odds ratio of 5-year asthma [2.07 (95%CI 1.43–3.01) *p* < .001] (*n* = 609/2,073) (Figure 1). In particular, systemic antibiotics taken for a respiratory indication (upper or lower respiratory) were associated with higher odds of asthma at the 5-year visit (aOR 2.36 [95%CI 1.59–3.48] *p* < .001 (*n* = 477/1,941) (Figure 1). However, children who received systemic antibiotics for only non-respiratory indications by 18 months were not associated with increased odds of asthma [aOR 1.08 (95%CI 0.44–2.30) *p* = .851] (*n* = 132/1,596).

**Figure 2:**
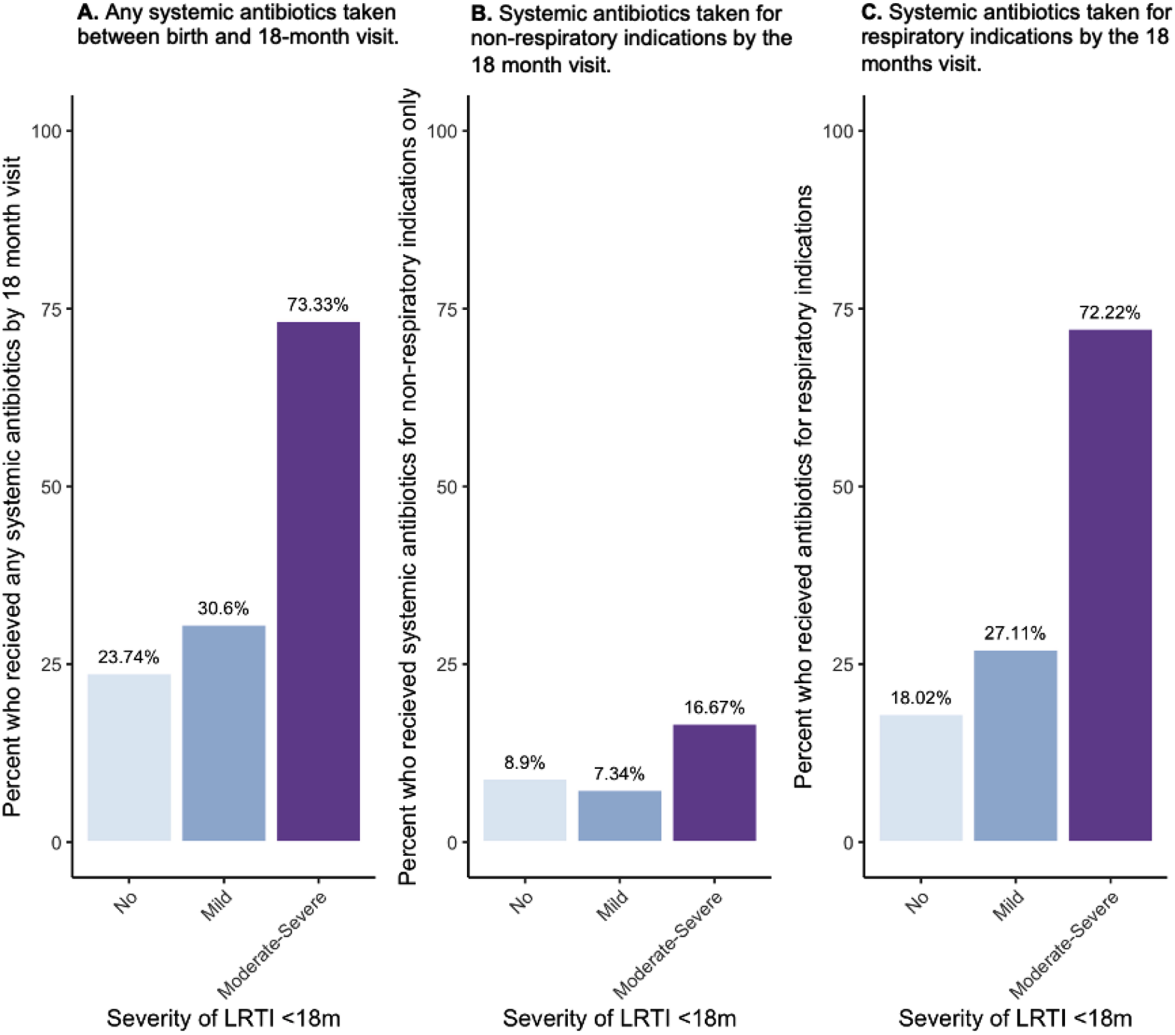
Percent of CHILD study participants who had taken systemic antibiotics between birth and the 18-month visit by indication (any reason, only non-respiratory, any respiratory) and by severity of LRTIs reported by 18 months of age. **(A)** Percent (%) of participants who received any systemic antibiotic across LRTI severities, **(B)** percent of participants who took antibiotics due to only non-respiratory indications across LRTI severities, **(C)** percent of participants who took antibiotics by the 18-month visit due to respiratory indications by LRTI severity group.

Next, we explored the association between severity of LRTI and history of early-life antibiotics. We observed that a higher portion of participants with a history of moderate-severe LRTI in the first 18 months had received systemic antibiotics during that period (73%) compared to mild (30.6%) and no LRTI participants (23.7%) (Figure 2A). Children with moderate-severe infections (defined here as requiring unscheduled healthcare services) more often received systemic antibiotics during early life, both for non-respiratory indications (only) (16.7% vs. Mild; 7.3% and No; 8.9%) (Figure 2B) as well as for respiratory indications (72% of Moderate-Severe LRTI participants vs. 27.11% of Mild and 18.02% of No LRTI) (Figure 2C).

### Antibiotics mediate the relationship between moderate-severe LRTI and 5-year asthma

We next explored the combined associations between LRTIs and systemic antibiotics during the first 18 months of life on 5-year asthma diagnosis. LRTI under 18 months were significantly associated with systemic antibiotics taken during that period [aOR 1.60 (95%CI 1.32–1.95) *p* < .001](Table 2). In combined models of any LRTI and any systemic antibiotics under 18 months, only antibiotics were significantly associated with increased odds of 5-year asthma [aOR 2.00 (95%CI 1.37–2.90) *p* = .002] (Table 2). Since LRTIs were not significantly associated with asthma in combined models with antibiotics (*p* = .08) (Table 2), mediation analyses focused on moderate-severe infections.

**Table 2:** Individual and combined regression models analyzing the association between any LRTIs within the first 18 months of life (LRTI <18 m) or the severity of those LRTIs (LRTI severity) and the use of systemic antibiotics for any indication before the 18-month visit, on the odds of an asthma diagnosis at age 5.

	Adjusted LRTI <18 m model^a^			Adjusted LRTI severity model^a^			
	(*N* = 2,073)			(*N* = 2,073)			
	aOR	CI	*p*		aOR	CI	*p*
Crude models: asthma 5Y∼X							
Asthma 5Y∼LRTI < 18 m				Asthma 5Y∼LRTI severity			
LRTI < 18 m				LRTI severity			
Yes	1.50	1.04–2.20	**0.033**	Mild	1.34	0.91–1.98	0.145
–				Moderate-severe	4.12	2.04–7.95	**<0.001**
Asthma 5Y∼1 + antibiotics				Asthma 5Y–1 + antibiotics			
Any systemic antibiotics	2.07	1.43–3.01	**<0.001**	Any Systemic Antibiotics	1.87	1.26–2.74	**0.002**
Mediator model: antibiotics (M)∼LRTI <18 m(X)				Mediator model: antibiotics (M)∼LRTI severity (X)			
Antibiotics∼1 + LRTI < 18 m				Antibiotics∼1 + LRTI severity			
LRTI < 18 m				LRTI severity			
Yes	1.60	1.32–1.95	**<0.001**	Mild	1.41	1.15–1.72	**0.001**
–				Moderate-severe	9.05	5.37–15.85	**<0.001**
Outcome model: 5-year asthma (Y)∼antibiotics (M) + LRTI < 18 m (X)				Outcome model: 5-year asthma (Y)∼antibiotics (M) + LRTI severity (X)			
LRTI < 18 m				LRTI severity			
Yes	1.40	0.96–2.06	0.080	Mild	1.29	0.86–1.95	0.216
–				Moderate-severe	3.36	1.59–6.81	**0.001**
Any antibiotics	2.00	1.37–2.90	**<0.001**	Any antibiotics	1.82	1.21–2.72	**0.004**

Bold indicates significant *p* values.

^a^ Models adjusted by: site, child sex, Caucasian, breastfeeding <3 m, prenatal smoke exposure, mode of delivery, family income, away from home <12 m, older siblings, and parental history of asthma.

In combined mutually adjusted models of LRTI severity and any systemic antibiotics taken before the 18-month visit, both moderate-to-severe LRTIs [aOR 3.36 (95%CI 1.59–6.81) *p* = .001] and systemic antibiotics were significantly associated with 5-year asthma [aOR 1.82 (95%CI 1.21–2.72) *p* = .004] (Table 2). Since both moderate-severe LRTI and systemic antibiotics were associated with increased risk of asthma, we explored the potential mediating effect of systemic antibiotics taken for any indication.

**Figure 3:**
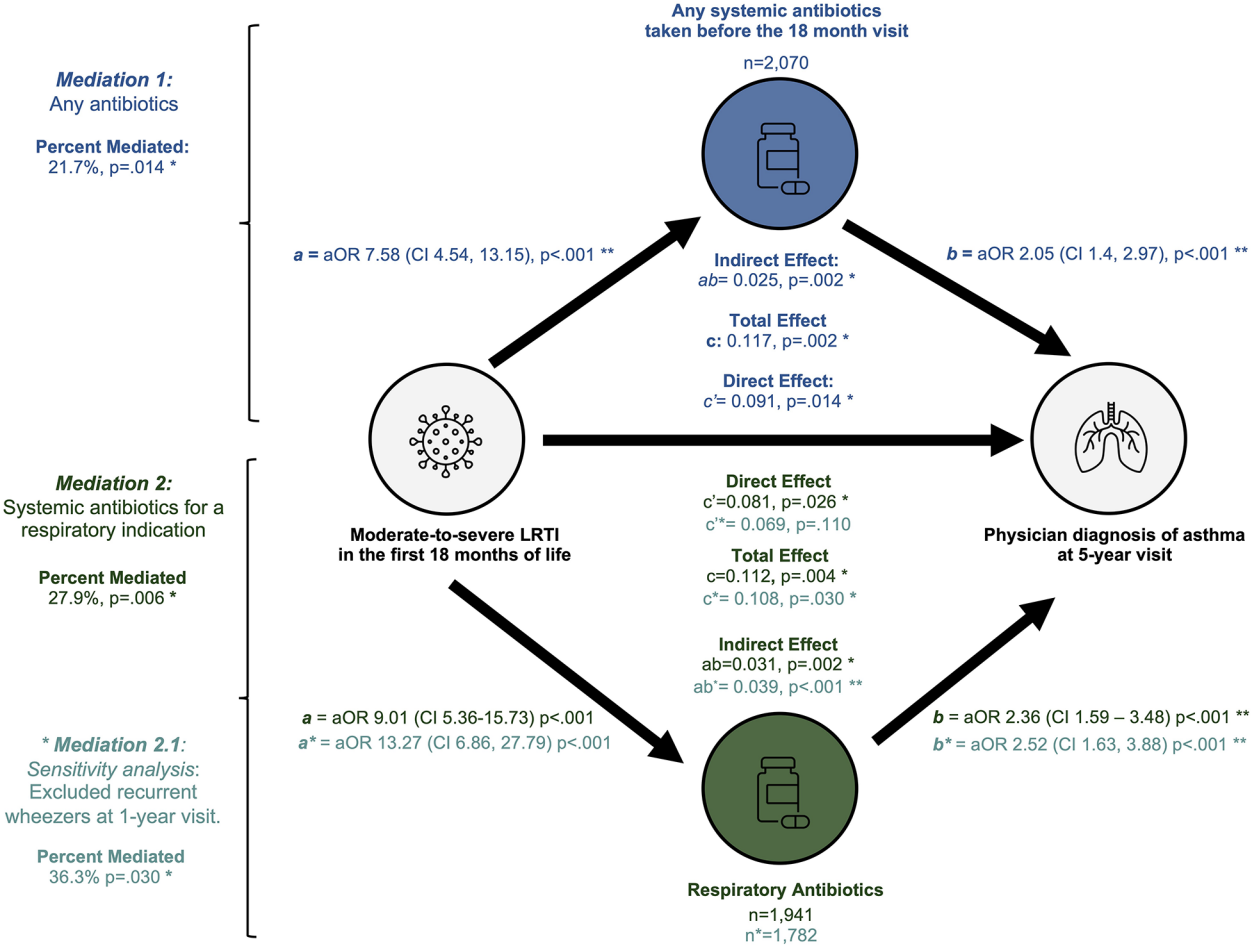
Summary of mediation analyses. This figure illustrates the three mediation models performed: any systemic antibiotics (top), antibiotics for any respiratory indication (second from bottom) and respiratory indication among non-wheezers (bottom).

In our first mediation analysis we estimated that 21.7% (*p* = .004) of the association between moderate-to-severe LRTIs and 5-year visit asthma was mediated through use of systemic antibiotics [β_indirect effect_ = 0.025 (95%CI 0.008–0.050), *p* = .002] (*n* = 2,070) (Table 3, Figure 3). A direct effect of moderate-to-severe LRTIs on 5-year asthma remained significant in this model [β_direct effect_ = 0.091 (95%CI 0.0019–0.170), *p* = .014] (Table 3, Figure 3).

**Table 3:** Mediation 1: systemic antibiotics taken for any indication as a mediator between severe LRTI and 5-year asthma (*N* = 2,070).

Adjusted model^a^ (*N* = 2,070)				
	OR (95% CI)			*p*-value
5-year asthma^a^∼X				
Moderate-to-severe LRTI (vs. mild & no)	3.51 (1.79, 6.46)			**<** **.** **001**
Any systemic antibiotics	2.05 (1.4, 2.97)			**<**.**001**
Mediator model: systemic antibiotics by 18 months ∼LRTI severity				
Moderate-to-severe LRTI	7.58 (4.54, 13.15)			**<**.**001**
Outcome model: asthma 5Y∼LRTI severity + systemic antibiotics by 18 months				
Moderate-to-severe LRTI	2.79 (1.39, 5.26)			.**002**
Any systemic antibiotics	1.83 (1.24, 2.68)			.**002**
Mediation output	β	95% CI lower	95% CI upper	*p*
Total effect	0.117	0.039	0.210	.**002**
Indirect effect (ab)	0.025	0.008	0.050	.**002**
Direct effect (c’)	0.091	0.019	0.170	.**014**
Proportion mediated,%	21.7%	7.7	54.0	.**004**

Bold indicates significant *p* values.

^a^ Models adjusted by: site, child sex, Caucasian, breastfeeding <3 m, prenatal smoke exposure, mode of delivery, family income, away from home <12 m, older siblings, and parental history of asthma.

### Indication for antibiotics and early-life recurrent wheeze alter the mediating role of systemic antibiotics on asthma

We observed a stronger association between 5-year asthma and early systemic antibiotics used for respiratory indications (Figure 1) and therefore replicated our mediation models after stratifying participants by indication. First, we examined the mediating role of antibiotics taken for a respiratory indication on the association between early-life LRTIs and 5-year asthma. Similar associations for moderate-severe LRTI [aOR 2.85 (95%CI 1.33–5.82), *p* = .005] and systemic antibiotics for any respiratory indication remained with 5-year asthma [aOR 2.05 (95%CI 1.36–3.08), *p* = .001] among children who received antibiotics for any respiratory indication (*n* = 1,941) (Supplementary Table 4). Significant mediation of the association between moderate-severe LRTIs and 5-year asthma (27.7%, *p* = .006) remained among children who took systemic antibiotics for any respiratory indications [β_indirect effect_ = 0.031 (95%CI 0.015–0.050), *p* = .002] (Supplementary Table 5). A significant direct effect of moderate-severe LRTI on risk of 5-year asthma remained among these participants [β_direct effect_ = 0.081 (95%CI 0.011–0.160), *p* = .026] (Supplementary Table 5).

Next, we explored whether the association between systemic antibiotics for a respiratory indication and asthma may be an artefact of children with pre-existing wheeze symptoms. We performed a sensitivity mediation in a subset of *n* = 1,748 participants who did not have recurrent wheezing already present at the 1-year visit (Supplementary Table 6, Figure 3). Mediation analysis in non-wheezers estimated that 36.3% (*p* = .030) of the relationship between moderate-to-severe LRTIs before 18 months and asthma at age 5 was mediated through systemic antibiotics taken for a respiratory indication [β_indirect_ = 0.039 (95%CI 0.015–0.060), *p* < .001]. However, there was no significant direct effect of moderate-severe LRTI in the non-recurrent wheezing subset [β_direct_ = 0.069 (95%CI −0.015 - 0.160), *p* = .110] (Supplementary Table 7, Figure 3).

Finally, systemic antibiotics used between birth and 18 months for only non-respiratory indications was not associated to 5-year asthma in this analysis [aOR 1.08 (95%CI 0.44–2.30), *p* = .851] (Supplementary Table 7). Mediation analysis in this subset of participants was not pursued due to the limited number of participants with moderate-severe LRTI and antibiotics taken in infancy for only non-respiratory indications (*n* = 8).

## Discussion

Using data collected in the CHILD Study we investigated the role of LRTIs and systemic antibiotics in the first 18-months of life on asthma diagnosis at the 5-year visit. While in our adjusted models the presence of any LRTI in the first 18 months was not a significant risk factor for asthma, we found that moderate-severe LRTIs significantly increased the estimated risk of asthma at age 5. Through mediation analyses we estimated that over 20% of this association (moderate-severe LRTIs and asthma) may be attributed to systemic antibiotic exposure, particularly among children who were prescribed antibiotics for a respiratory indication. Moreover, these associations were present among children without wheezing symptoms in the first year of life and preceding symptoms of early life asthma.

We provide evidence that systemic antibiotics taken in the first 18 months of life, regardless of the indication, partially mediates the association between moderate-severe LRTI and 5-year asthma. In subsequent mediation analyses, we identified that the proportion mediated was increased in cases where the systemic antibiotics were taken for respiratory indications. Suggesting a direct association between infection severity on asthma, independent of, yet compounded by, antibiotic use. In our sensitivity analysis in only non-recurrent wheezers (excluding children with recurrent wheezing at 1-year visit), the mediating effect of respiratory antibiotics was increased even further, with no significant direct effect of moderate-severe LRTIs remaining. These results suggest that in children without early symptoms of asthma, a substantial proportion of the association between moderate-severe LRTIs and asthma may be explained by systemic antibiotic exposure rather than a respiratory infection itself.

Our study is the first to investigate the mediating role of antibiotic use in the association between early-life LRTIs and asthma at age 5. Nonetheless, our results support observations from other studies. Bentouhami et al. (17) performed an incidence density study nested in a data collection project with information on 1,128 mother–child pairs where systemic antibiotic use in the first year of life was defined as excessive (≥4 courses) vs. non-excessive (<4 courses) use based on information from weekly diaries (17). The authors found a stronger association between the incidence of asthma and the use of systemic antibiotics in the first year of life among children who had LRTIs (defined as having had bronchitis with or without chronic cough and/or pneumonia according to the reporting of the parents) during that time. The asthma incidence density ratio (IDR) was significantly higher for children with LRTIs (IDR 5.17) compared to those without (IDR 1.49). Similarly, the Longitudinal Study of Australian Children (LSAC) found that antibiotics given to children between birth and 24 months increased their risk of developing asthma later in childhood (6 and 15 years old) using medication record data (18). After LSAC authors accounted for respiratory infections that prompted antibiotic use (such as ear infections, hospitalization for fever or viral infections in the first year of life), early antibiotic use in children still significantly increased the risk of developing early-persistent asthma by 2.3 times (95% CI: 1.47–3.67, *p* < 0.001) compared to those without antibiotic exposure (18). Finally, a systematic review demonstrated that after adjusting for respiratory infections, there was still a significant and independent association between antibiotics and asthma (although decreasing from OR 1.38 to OR 1.16 after adjustment) (19).

Several mechanisms have been proposed to explain the association between LRTIs, antibiotics, and asthma. One hypothesis suggests that antibiotics may eliminate beneficial bacteria, which otherwise have a protective effect, thereby increasing the risk of allergic illnesses. Another possibility is that antibiotics with anti-inflammatory properties, such as macrolides, may inhibit type 1 immune responses, leading to a dominance of type 2 immune responses. Antibiotics might also contribute to the development of asthma by disrupting the human microbiome in a critical period when the human microbiome and immune system are developing (20–22). It is hypothesized that this effect is caused through perturbations in the populations, community succession, and diversity of the airway and gut microbiota. Using >2 antibiotic treatments between birth and 11 months of age was associated with an increased risk of asthma which was partially mediated through longitudinal change in the composition of the nasal microbiome (12). Infant antibiotic use was associated with elevated *Moraxella, Haemophilus,* and *Streptococcus,* which are pathogenic associated genus (23) and high *Moraxella sp.* abundance is associated with preschool asthma (12, 24). There is also evidence that antibiotics increase the risk of asthma through their influence on the gut microbiome (25–27) thought to occur through depletion of beneficial bacteria with fermentative capacity (e.g., short chain fatty acid producing species), decreased overall diversity and therefore reduced resistance to pathogenic bacteria colonization (28). These microbial disruptions can exacerbate airway inflammation or may impact proper immune system development increasing the risk of developing asthma. In the CHILD Study, the number of antibiotics courses within the first year of life was associated with decreased gut alpha diversity, and this effect was greatest when taken under 3 months of age (11). CHILD authors further demonstrated that the gut microbiome diversity at 1 year mediated the relationship between infant antibiotic use and asthma diagnosis at age 5 (11).

In our analyses we followed the most widely used mediation methodology outlined by Baron and Kenny (11) which requires the following assumptions to be satisfied: (i) no misspecification of causal order, (ii) no misspecification of causal direction, (iii) no misspecification due to unmeasured variables, and (iv) no misspecification due to imperfect measurement (29). By measuring and defining our variables in terms of temporality and having a theory for biological plausibility we minimize risks of violating the first two assumptions. However, as an observational study it is not possible to guarantee complete adherence of the latter two assumptions. We acknowledge that our reliance on parental reporting may overestimate LRTI prevalence. We attempted to mitigate this by including fever as a required factor in our symptom-based definition of LRTIs. Fever is an objective indicator of symptomatic infection that parents can measure; inclusion of fever in our symptom-based definition is supported by our validation analysis of LRTI symptoms vs. virological testing, where including fever significantly improved the identification of true positives (laboratory confirmed LRTIs). The trade-off between the increased specificity of objective diagnostic tests and decreased applicability in primary care or low-resource settings needs to be evaluated in the design of future research studies.

In this context, our classification of infection severity using healthcare utilization may also be biased by parental behavior. Parents with heightened vigilance due to increased medical literacy or family history of asthma may be more inclined to seek medical care. Such patterns could influence our classification of LRTI severity and the likelihood of receiving antibiotics. However, our use of a validated LRTI definition ensures that the classification is based on consistent, clinically relevant criteria rather than subjective or behavioral factors, improving the reliability of our assessment. Additionally, we attempted to minimize these parental factors by adjusting our models for family income and family history of asthma. Our symptom-based LRTI definition may be used in future population-based studies to explore the associations reported here and could be adapted for other parental reported studies, data-linkage studies, or in low-income settings where objective testing for infection may not be readily available.

Given that our LRTI and antibiotic data rely on parental reports collected through questionnaires administered at regular intervals, we could not report on associations outside these intervals, such as the impact of earlier or later timing of infections or antibiotics during the first 18 months, or the cumulative antibiotic exposure in infancy. Nonetheless, findings from a smaller subset of CHILD Study participants that linked administrative data found a significant dose-response between number of antibiotic courses and asthma, with other studies reporting similar findings (11, 18, 30). While we were unable to replicate this approach in our mediation models due to sample size limitations, this gap highlights an area for future investigation, particularly concerning the hypothesis that earlier-timing of infections and frequent antibiotic treatment could be more detrimental to the development of the microbiome and immune system. Imperfect measurement of asthma in this age group may also be possible. Differentiating early signs of asthma from symptoms of early respiratory infections is a complex methodological challenge. In young children, asthma is often used as an umbrella diagnosis that is not well defined. As a result, the classification of pediatric asthma may be susceptible to bias, with certain subtypes potentially being underrepresented or overrepresented.

Another limitation to our mediation methodology is that any interactions between LRTIs and type of antibiotics cannot be tested. We attempted to explore this question using an alternative approach, by conducting stratified analysis by antibiotic indication (31). Among children who had received respiratory antibiotics only, we estimated a significant mediating effect of 21.7%. Unfortunately, we were unable to replicate this analysis in children who had received only non-respiratory antibiotics due to insufficient sample size in this mediator group, a challenge that would have persisted in a formal interaction analysis. This limited sample size likely reflects the high co-occurrence of LRTIs and antibiotics during this period, where many LRTIs were treated with antibiotics. As the CHILD Study is a general population cohort, the small number of participants with moderate-to-severe LRTIs treated exclusively with non-respiratory antibiotics further constrained our analysis. Additionally, our classification of antibiotics into “any respiratory” and “non-respiratory” indications complicates categorization, as children may receive antibiotics for multiple indications. To address this, and in line with our primary objective, we included children who received antibiotics for both indications in the “any respiratory antibiotic” group, capturing instances where antibiotics for LRTI could mediate the respiratory pathway to asthma. However, interpreting these findings requires caution, as attributing risk solely to respiratory antibiotics may overestimate their effect or underestimate the effect of non-respiratory antibiotics.

Finally, a limitation of this mediation approach is the categorical nature of our mediator and outcome, which in part is due to non-collapsibility in logistic regression models and may lead to underestimation of proportions mediated (32). Where the mediator can effectively be intervened upon, and where assumptions of consistency, exchangeability, and positivity are complied with, future studies should consider implementation of causal mediation analysis using a potential outcomes framework to estimate controlled direct and indirect effects (33). Future studies may also benefit from evaluating the confounding role of factors not measured here such as genetic background, epigenetic effects and airway microbiome interactions. Our study design is also at risk of confounding by indication where severe LRTIs requiring antibiotics may be linked to future asthma diagnoses due to shared underlying susceptibilities such as impaired lung function, genetic predisposition to asthma or early immune dysregulation that increase risk of both early-life LRTIs and later asthma. This makes it challenging to seperate the effect of antibiotics from the effect of the infection, and as a result, our model may overestimate the contribution of systemic antibiotics to the development of asthma. To minimize this, following pharmacoepidemiologic practices when confounding by indication cannot be directly measured, we did not include wheezing symptoms in our definition of LRTI, and conducted sensitivity analysis in non-recurrent wheezers at age 1 (34). Our findings remained consistent in these analyses, suggesting the association between antibiotics and asthma is not soley driven by misclassified early asthma/wheezing.

We also recognize that children living in remote areas, or from lower socioeconomic backgrounds experience a disproportionate burden of infection and asthma (35, 36). We address potential confounding of this nature by adjusting all analyses by factors such as study site and family income. Nonetheless, given that our definition of infection severity is based on healthcare utilization, future studies on populations with limited access to care are needed to assess the generalizability of our findings to these groups of children. While our study focuses on the impact of early-life antibiotic use in the general population, it is crucial to recognize that high-risk patient populations or cases of severe infections require distinct clinical considerations, and we do not advocate for withholding antibiotics in cases when they are necessary. Additionally, given the diversity of healthcare systems worldwide, further research in settings outside Canada is needed to assess the generalizability of our findings to populations with differing healthcare practices and needs. Finally, considering that antibiotic prescriptions from our study occurred during the 2010–2012 period, prescribing practices may have since evolved due to initiatives like “Choosing Wisely Canada”, potentially leading to fewer infants receiving antibiotics for viral LRTIs. Future studies should attempt to replicate these associations using current datasets to account for changes in clinical practices and to validate these findings in a contemporary cohort.

Overall, our study provides novel evidence that both the severity and management of the infection, rather than simply the occurrence of a LRTI *per se*, may be relevant to asthma development. This highlights the importance of prudent antibiotic use, especially considering the potential long-term effects of these treatments and contribution of unnecessary use to rising antibiotic resistance. While our study illuminates potential links and mechanisms, it also emphasizes the complexity of asthma development and the multifaceted influence of early-life exposures. Ultimately, our results are based on observational data, and the significance of antibiotic use as a mediator in this study does not necessarily confirm causality. There is need for further research involving a finer examination of types of respiratory infection, severity of infection, and nature of antibiotic usage to elucidate these relationships and inform strategies to mitigate risk of developing asthma.

## Data Availability

The data analyzed in this study is subject to the following licenses/restrictions: data from the CHILD Cohort Study is available to researchers upon request. Requests to access these datasets should be directed to https://childstudy.ca/for-researchers/study-data.
