# Supplementary material for: Exploring the potential mediating role of systemic antibiotics in the association between early-life lower respiratory tract infections and asthma at age 5 in the CHILD study
Source: Front Allergy. 2025 Jan 21;5:1463867. doi: 10.3389/falgy.2024.1463867 (PMC11790586; doi:10.3389/falgy.2024.1463867)
Supplement: Supplementary file 1 [file Supplementaryfile1.docx]

Supplementary Material

**Exploring the potential mediating role of systemic antibiotics in the association between early-life lower respiratory tract infections and asthma at age 5 in the CHILD study**

Medeleanu MV, Reyna ME, Dai DLY, Winsor GL, Brinkman FSL, Verma R, Nugent E, Riaz N, Simons E, Mandhane PJ, Azad MB, Turvey SE, Moraes TJ and Subbarao P*****

**Correspondence:** Corresponding Author: padmaja.subbarao@sickkids.ca

# Supplementary Figures and Tables

##
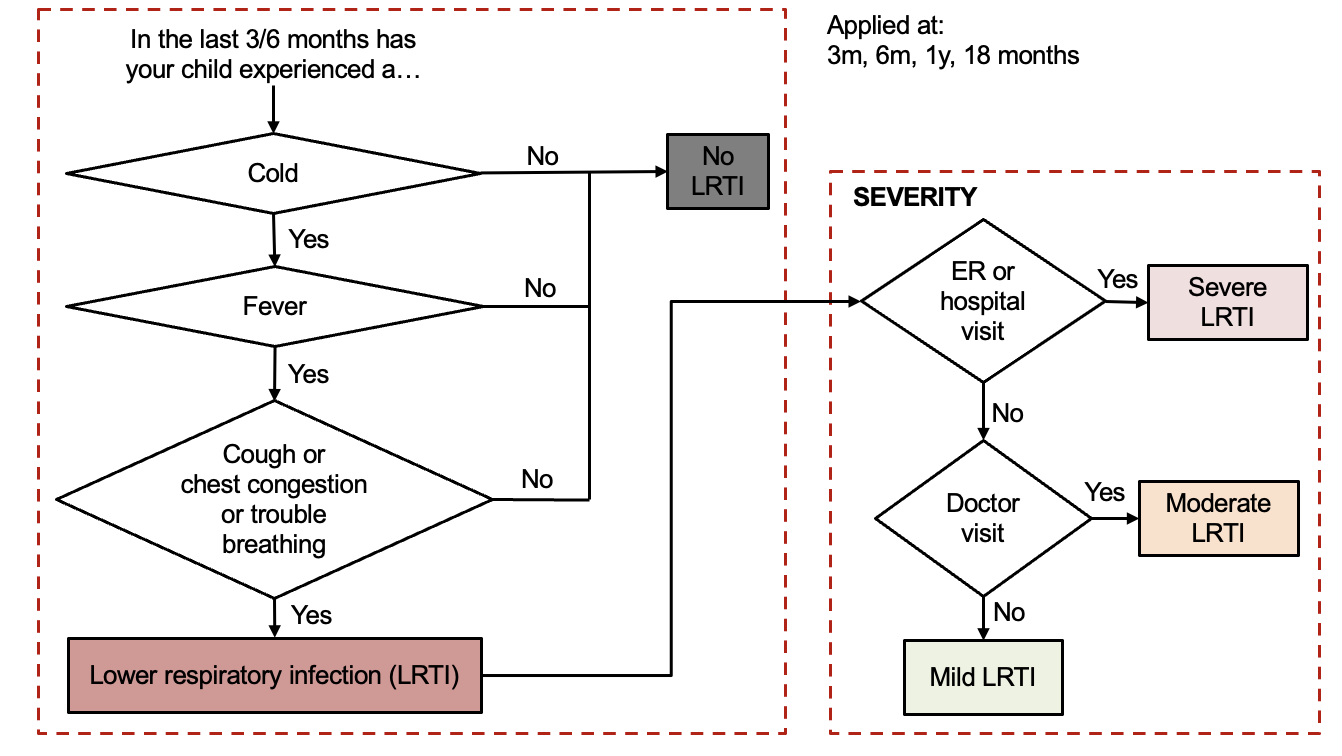


## Supplementary Figure 1. Epidemiological Lower Respiratory Tract Infection Definition flow chart. History of respiratory infections were assessed by parent reported questionnaires collected at 3, 6, 12 and 18 months of age regarding respiratory tract symptoms and health care utilization.


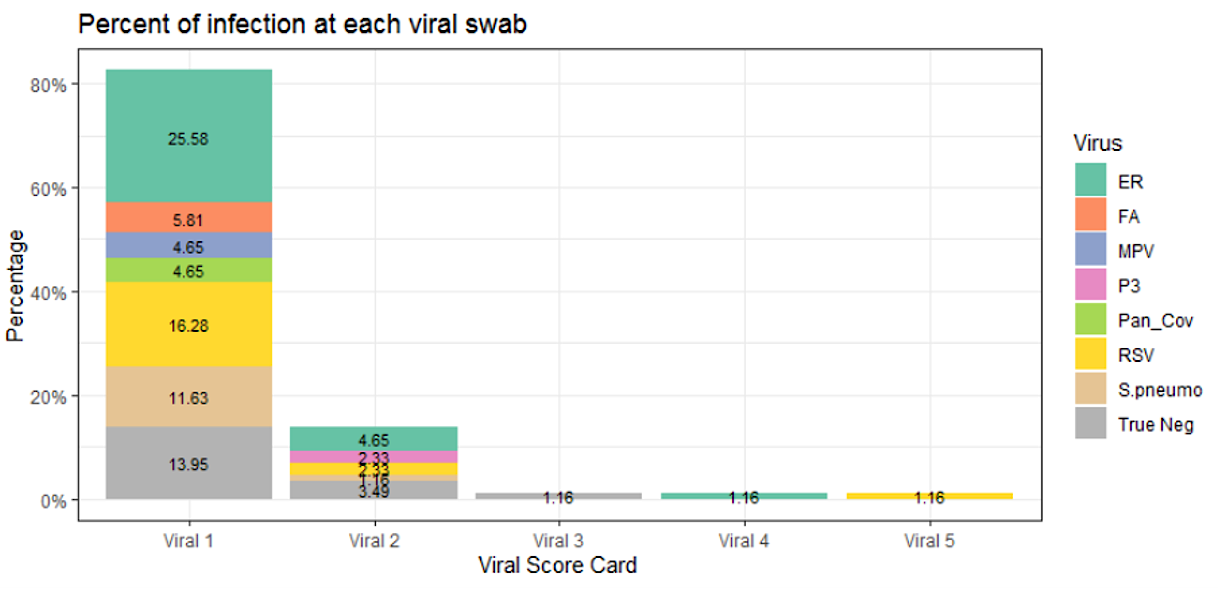


**Supplementary Figure 2.** Distribution of positive swabs for viruses and bacteria at each Viral Score Card call. Abbreviations: ER= Enterovirus/Rhinovirus, FA= Adenovirus FA, MPV= Metapneumovirus, P3= Parainfluenza 3, Pan_Cov= Coronaviruses, RSV= Respiratory syncytial virus, S.pneumo= Streptococcus pneumoniae and True Neg= Negative Swab.

**Supplementary Table 1.** Prevalence of symptoms reported in Viral Scorecards in the subset of participants by nasal swab result (negative or positive). Positive indicates positive for any virus only but excludes positive swabs for S. pneumonia.

| ​ Symptom Reported | Negative swab​  (n=25) | Positive swab  (n=56) | p​ |
| --- | --- | --- | --- |
| *Any Wipe Nose (%)​* | 3 (50.0)​ | 19 (55.9)​ | 1.000​ |
| *Any Fever (%)​* | 1 (33.3)​ | 7 (53.8)​ | 1.000​ |
| *Any Difficulty Breathing (%)​* | 4 (40.0)​ | 22 (57.9)​ | 0.513​ |
| *Any Stuffy Nose (%)​* | 3 (75.0)​ | 21 (100.0)​ | 0.344​ |
| *Any Cough in the Last 3 Days (%)​* | 10 (83.3)​ | 37 (100.0)​ | 0.090​ |
| *Any Runny Nose (%)​* | 6 (50.0)​ | 36 (92.3)​ | 0.003​ |
| *Any Wheeze or Whistle (%)​* | 1 (10.0)​ | 11 (35.5)​ | 0.254​ |
| *Any Ribs Drawn In (%)​* | 1 (25.0)​ | 2 (10.5)​ | 1.000​ |
| *Any Rapid Breathing (%)​* | 2 (50.0)​ | 3 (15.8)​ | 0.400​ |
| *Any Bluish Features (%)​​* | 0 (0.0)​ | 0 (0.0)​ | -​ |


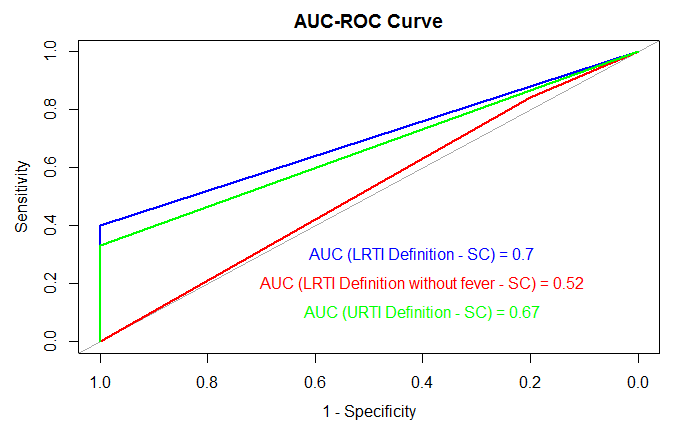


**Supplementary Figure 3.** Area under the receiver operator curves (AUROC) depicting performance of LRTI and URTI definition with and without fever applied to the viral scorecard results, cross referenced with swab results. 12 swabs were included in LRTI Definition vs Positive Swab Result ROC curve, 48 swabs included in LRTI w/o Fever vs Positive Swab Result ROC curve and 11 swabs included in URTI vs Positive Swab Result ROC curve.

**Supplementary Table 2.** Confusion Matrix Statistics for each CHILD Respiratory Infection Definition.

| CHILD Respiratory Infection Definitions​ | Area under receiver operating characteristic curve | 95% CI​ | Kappa Statistic​ | Sensitivity​ | Specificity​ | Positive Predictive Value | Negative Predictive Value |
| --- | --- | --- | --- | --- | --- | --- | --- |
| *LRTI definition* | 0.70 | (0.21, 0.79)​ | 0.18​ | 1.00​ | 0.40​ | 0.25​ | 1.00​ |
| *LRTI definition without fever​* | 0.52 | (0.56, 0.83)​ | 0.05​ | 0.20​ | 0.84​ | 0.25​ | 0.80​ |
| *URTI definition* | 0.67 | (0.17,0.77)​ | 0.15​ | 1.00​ | 0.33​ | 0.25​ | 1.00​ |

Participants with >1 LRTI questionnaire completed

n=3,301

CHILD participants assessed for inclusion

N=3,454

Participants with 5-year Asthma Diagnosis

n=2,388

Participants with medication history from birth to 18 months including mode (topical or oral) and indication (respiratory, non-respiratory symptom)

n=2,073

**Supplementary Figure 4**. Consort Diagram of CHILD Study Participants assessed for eligibility.


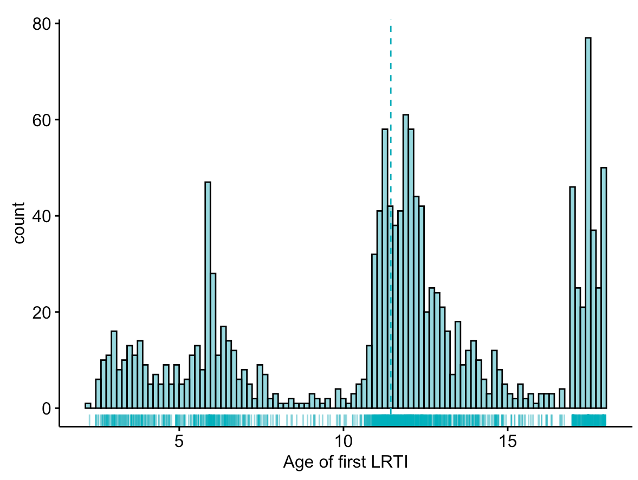


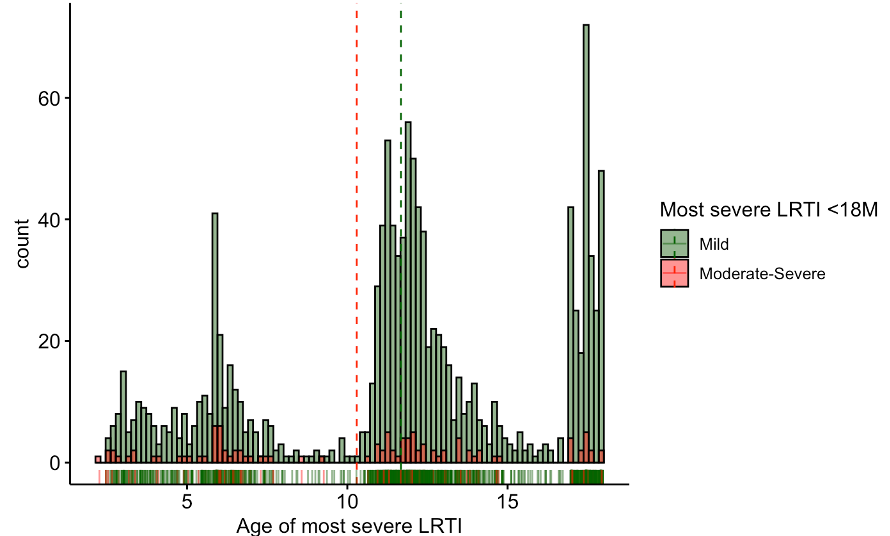


**Supplementary Figure 5**. Prevalence of (A) any LRTI during the first 18 months or (B) most severe LRTI during the first 18 months of life.

**Supplementary Table 3.** Demographic and clinical characteristics of CHILD participants in LRTI and LRTI Severity groups.

|  | CHILD Cohort | LRTI in the first 18 months | | | Severity of LRTIs in the first 18 months | | | |
| --- | --- | --- | --- | --- | --- | --- | --- | --- |
|  | (n=3,454) | No  (n=1881) | LRTI  (n=1420) | p | No  (n=1678) | Mild  (n=1490) | Moderate-Severe  (n=107) | p |
| **Child sex, Male (%)** | 1816 (52.6) | 946 (51.0) | 772 (54.4) | .067 | 857 (51.1) | 805 (54.0) | 60 (56.1) | .192 |
| **Caucasian, Yes (%)** | 2122 (64.6) | 961 (51.1) | 772 (54.4) | .082 | 1045 (63.9) | 969 (65.7) | 64 (60.4) | .392 |
| **Study Site (%)** |  |  |  | **.019** |  |  |  | **.003** |
| *Edmonton* | 812 (23.5) | 412 (21.9) | 331 (23.3) |  | 367 (21.9) | 352 (23.6) | 17 (15.9) |  |
| *Toronto* | 813 (23.5) | 476 (25.3) | 296 (20.8) |  | 438 (26.1) | 298 (20.0) | 29 (27.1) |  |
| *Vancouver* | 787 (22.8) | 439 (23.3) | 332 (23.4) |  | 380 (22.6) | 363 (24.4) | 25 (23.4) |  |
| *Winnipeg and surrounding areas* | 1043 (30.2) | 554 (29.5) | 461 (32.5) |  | 493 (29.4) | 477 (32.0) | 36 (33.6) |  |
| **Weight for age z-score (mean (SD))** | 0.28 (0.99) | 0.26 (1.01) | 0.32 (0.96) | .069 | 0.26 (1.00) | 0.30 (0.99) | 0.37 (0.82) | .375 |
| **Any Prenatal Smoke Exposure, yes (%)** | 610 (17.7) | 335 (17.8) | 250 (17.6) | .916 | 301 (17.9) | 261 (17.5) | 22 (20.6) | .72 |
| **Family Income at 18 weeks (%)** |  |  |  | .497 |  |  |  | .477 |
| *$0 - $49,999* | 414 (12.5) | 210 (11.6) | 184 (13.3) |  | 190 (11.7) | 182 (12.5) | 18 (17.3) |  |
| *$50,000 - $99,999* | 999 (30.1) | 550 (30.3) | 422 (30.5) |  | 491 (30.3) | 441 (30.4) | 33 (31.7) |  |
| *$100,000 - $149,999* | 835 (25.2) | 466 (25.6) | 347 (25.1) |  | 404 (24.9) | 383 (26.4) | 21 (20.2) |  |
| *> $150,000* | 746 (22.5) | 406 (22.3) | 307 (22.2) |  | 367 (22.6) | 317 (21.8) | 24 (23.1) |  |
| *Prefer not to say* | 323 (9.7) | 186 (10.2) | 124 (9.0) |  | 171 (10.5) | 128 (8.8) | 8 (7.7) |  |
| **Time away from home <18 m, yes (%)** | 1345 (55.7) | 671 (53.3) | 663 (58.5) | **.012** | 584 (52.9) | 686 (57.9) | 58 (62.4) | **.022** |
| **Older Siblings, Yes (%)** | 1452 (45.9) | 722 (41.1) | 683 (51.6) | **<.001** | 623 (40.9) | 722 (49.8) | 60 (57.1) | **<.001** |
| **Delivery Mode, Cesarian section (%)** | 845 (25.6) | 471 (25.4) | 359 (25.6 | .919 | 422 (25.5) | 371 (25.3) | 26 (24.5) | .976 |
| **Hospital Breastfeeding Status** |  |  |  | .792 |  |  |  | .787 |
| *Exclusive* | 2000 (72.0) | 1102 (72.2) | 838 (72.9) |  | 956 (71.6) | 919 (73.5) | 65 (73.0) |  |
| *Partial* | 684 (24.6) | 379 (24.8) | 274 (23.8) |  | 340 (25.5) | 292 (23.3) | 21 (23.6) |  |
| *Zero* | 94 (3.4) | 45 (2.9) | 37 (3.2) |  | 39 (2.9) | 40 (3.2) | 3 (3.4) |  |
| **3-month Breastfeeding** |  |  |  | .488 |  |  |  | .104 |
| *Exclusive* | 1884 (59.8) | 1077 (60.1) | 798 (59.3) |  | 937 (60.2) | 884 (59.9) | 54 (50.9) |  |
| *Partial* | 821 (26.1) | 456 (25.4) | 365 (27.1) |  | 391 (25.1) | 400 (27.1) | 30 (28.3) |  |
| *Zero* | 445 (14.1) | 260 (14.5) | 182 (13.5) |  | 228 (14.7) | 192 (13.0) | 22 (20.8) |  |
| **6-month Breastfeeding** |  |  |  | .738 |  |  |  | .608 |
| *Exclusive* | 552 (18.1) | 312 (18.3) | 236 (17.8) |  | 266 (18.1) | 268 (18.4) | 14 (13.2) |  |
| *Partial* | 1766 (58.1) | 979 (57.6) | 783 (59.0) |  | 849 (57.9) | 851 (58.4) | 62 (58.5) |  |
| *Zero* | 724 (23.8) | 410 (24.1) | 309 (23.3) |  | 352 (24.0) | 337 (23.1) | 30 (28.3) |  |
| **Inhalant Sensitization at age 1, Yes (%)** | 124 (4.1) | 67 (4.1) | 54 (4.0) | 1.00 | 57 (4.0) | 59 (4.1) | 5 (4.8) | .919 |
| **Atopy at age 1, Yes (%)** | 419 (13.8) | 228 (13.9) | 183 (13.6) | .876 | 204 (14.2) | 186 (13.0) | 18 (17.1) | .359 |
| **Atopy at age 3, Yes (%)** | 403 (14.2) | 216 (14.1) | 180 (14.3) | .903 | 191 (14.1) | 188 (14.3) | 17 (17.7) | .626 |
| **Atopy at age 5, Yes (%)** | 527 (19.5) | 286 (19.7) | 229 (19.0) | .674 | 248 (19.4) | 239 (18.9) | 26 (27.7) | .115 |
| **Recurrent wheeze at age 1, Yes (%)** | 259 (7.8) | 90 (4.8) | 169 (11.9) | **<.001** | 83 (5.0) | 136 (9.1) | 36 (33.6) | **<.001** |
| **Recurrent wheeze at age 3, Yes (%)** | 265 (8.8) | 121 (7.4) | 143 (10.8) | **.001** | 111 (7.7) | 127 (9.2) | 24 (22.9) | **<.001** |
| **Asthma Diagnosis at 3-year visit, yes (%)** | 177 (6.6) | 82 (5.6) | 93 (7.9) | **.017** | 74 (5.7) | 79 (6.4) | 20 (24.1) | **<.001** |
| **Asthma Diagnosis at 5-year visit, yes (%)** | 171 (6.1) | 78 (5.2) | 91 (7.3) | **.023** | 70 (5.3) | 79 (6.0) | 19 (19.6) | **<.001** |
| **Parental History of Asthma, Yes (%)** | 1077 (37.8) | 570 (35.7) | 493 (40.4) | **.012** | 507 (35.9) | 500 (39.0) | 46 (46.9) | **.039** |

**Supplementary Table 4.** Individual and combined regression models of any LRTI /or/ severity of LRTIs in the first 18 months of life and systemic antibiotics taken for a respiratory indication before 18 months visit for any indication on odds of asthma diagnosis at age 5 (n=1,941). Models adjusted by: site, child sex, caucasian, breastfeeding <3m, prenatal smoke exposure, mode of delivery, family income, away from home <12m, older siblings, and parental history of asthma.

|  | Adjusted LRTI <18m Model * | | | | |  | | Adjusted LRTI Severity Model * | | | | | | |
| --- | --- | --- | --- | --- | --- | --- | --- | --- | --- | --- | --- | --- | --- | --- |
|  | (n=1,941) | | | | |  | (n=1,941) | | | | | | | |
|  | OR | CI | | p |  | | | | OR | | CI | | p | |
| Crude models: Asthma 5Y ~ X | | | | | | | | | | | | | | |
| Asthma 5Y ~ 1 + LRTI<18m | | | | | | Asthma 5Y ~ 1 + LRTI Severity | | | | | | | |  |
| *LRTI<18m* | | | | | | *LRTI Severity* | | | | | | | |  |
| Yes | 1.50 | | 1.02 – 2.23 | **0.039** | | Mild | | | 1.34 | 0.90 – 2.01 | | 0.154 | |  |
| - |  | |  |  | | Mod-Severe | | | 4.13 | 1.99 – 8.13 | | **<0.001** | |  |
| Asthma 5Y ~ 1 + Respiratory Antibiotics | | | | | | Asthma5Y ~ 1 + ATBX | | | | | | | |  |
| Respiratory Antibiotics <18m | 2.36 | | 1.59 – 3.48 | **<0.001** | | Respiratory Antibiotics <18m | | | 2.36 | 1.59 – 3.48 | | **<0.001** | |  |
| Mediator model:  Respiratory Antibiotics (M) ~ LRTI severity (X) | | | | | | Mediator model:  Respiratory Antibiotics (M) ~ LRTI severity (X) | | | | | | | |  |
| ATBX ~ 1 + LRTI<18m | | | | | | ATBX ~ 1 + LRTI Severity | | | | | | | |  |
| *LRTI<18m* | | | | | | *LRTI Severity* | | | | | | | |  |
| Yes | 1.95 | | 1.57 – 2.43 | **<0.001** | | Mild | | | 1.68 | 1.35 – 2.11 | | **<0.001** | |  |
| - |  | |  |  | | Mod-Severe | | | 12.09 | 7.07 – 21.44 | | **<0.001** | |  |
| Outcome model:  5-year Asthma (Y) ~ Resp. Antibiotics (M) + LRTI<18m (X) | | | | | | Outcome model:  5-year Asthma (Y) ~ Resp. Antibiotics. (M) + LRTI severity (X) | | | | | | | |  |
| *LRTI<18m* | | | | | | *LRTI Severity* | | | | | | | |  |
| Yes | 1.35 | | 0.91 – 2.01 | 0.138 | | Mild | | | 1.24 | 0.83 – 1.87 | | 0.290 | |  |
| - |  | |  |  | | Mod-Severe | | | 2.85 | 1.33 – 5.82 | | **0.005** | |  |
| Respiratory Antibiotics <18m | 2.25 | | 1.51 – 3.33 | **<0.001** | | Respiratory  Antibiotics <18m | | | 2.05 | 1.36 – 3.08 | | **0.001** | |  |

**Supplementary Table 5.** Mediation of moderate-severe LRTIs by 18 months of age on 5-year asthma by antibiotics for respiratory indications (n=1,941). *Adjustment set: site, child sex, ethnicity, breastfeeding status up to 3 months, prenatal smoke exposure, mode of delivery, family income, time spent away from home before the age of 12 months, presence of older siblings, and a parental history of asthma.

|  | **Adjusted Model ***  **(n=1,941)** | | | |
| --- | --- | --- | --- | --- |
|  | OR (95% CI) | | | p |
| 5-year Asthma ** ~ X |  |  |  |  |
| Moderate-to-Severe LRTI | 3.50 (1.75, 6.58) | | | **<.001** |
| Systemic ATBX for respiratory indication | 2.36 (1.59, 3.48) | | | **<.001** |
| Mediator model:  Respiratory antibiotics by 18 months ** ~ LRTI severity | | | | |
| Moderate-to-Severe LRTI | 9.01 (5.36, 15.73) | | | **<.001** |
| Outcome model:  Asthma 5Y~ LRTI severity + Respiratory Antibiotics by 18 months | | | | |
| Moderate-to-Severe LRTI | 2.49 (1.21, 4.82) | | | **.009** |
| Respiratory ATBX <18m | 2.11 (1.4, 3.15) | | | **<.001** |
| Mediation output: | β | 95% CI Lower | 95% CI Upper | p |
| Total effect | 0.112 | 0.038 | 0.200 | **.004** |
| Indirect effect (ab) | 0.031 | 0.015 | 0.050 | **.002** |
| Direct effect (c’) | 0.081 | 0.011 | 0.160 | **.026** |
| Proportion mediated, % | 27.9% | 12.5% | 73.0% | **.006** |

**Supplementary Table 6.** Mediation analysis of antibiotics for respiratory indication on 5-year Asthma among non-wheezers in the CHILD Study (n=1,782). *Adjustment set: site, child sex, ethnicity, breastfeeding status up to 3 months, prenatal smoke exposure, mode of delivery, family income, time spent away from home before the age of 12 months, presence of older siblings, and a parental history of asthma.

|  | **Adjusted Model ***  **(1,782)** | | | |
| --- | --- | --- | --- | --- |
|  | OR (95% CI) | | | p |
| 5-year Asthma ** ~ X |  | | |  |
| Moderate-to-Severe LRTI | 3.59 (1.49, 7.74) | | | **.002** |
| Systemic ATBX for respiratory indication | 2.52 (1.63, 3.88) | | | **<.001** |
| Mediator model:  Respiratory antibiotics by 18 months ** ~ LRTI severity | | | | |
| Moderate-to-Severe LRTI | 13.27 (6.86, 27.79) | | | **<.001** |
| Outcome model:  Asthma 5Y~ LRTI severity + Respiratory Antibiotics by 18 months | | | | |
| Moderate-to-Severe LRTI | 2.3 (0.92, 5.16) | | | .056 |
| Respiratory ATBX <18m | 2.29 (1.45, 3.57) | | | **<.001** |
| Mediation output: | β | 95% CI Lower | 95% CI Upper | p |
| Total effect | 0.108 | 0.013 | 0.210 | **.030** |
| Indirect effect (ab) | 0.039 | 0.015 | 0.060 | **<.001** |
| Direct effect (c’) | 0.069 | -0.015 | 0.160 | .110 |
| Proportion mediated, % | 36.3% | 12% | 140% | **.030** |

**Supplementary Table 7.** Individual and combined models of any LRTI /or/ severity of LRTIs in the first 18 months of life and non-respiratory systemic antibiotics taken by 18-month visit on odds of asthma diagnosis at age 5 (n=1,596). Models adjusted by: site, child sex, Caucasian, breastfeeding <3m, prenatal smoke exposure, mode of delivery, family income, away from home <12m, older siblings, and parental history of asthma.

|  | **Any LRTI in the first 18 months** | | |  | | **Severity of LRTI in the first 18 months** | | | |
| --- | --- | --- | --- | --- | --- | --- | --- | --- | --- |
|  | **Adjusted Model *** | | | | **Adjusted Model *** | | | | |
|  | **(n=1,596)** | | | **(n=1,596)** | | | | | |
|  | OR | CI | p | |  | | OR | CI | p |
| Crude models: Asthma5Y ~ X | | | | | | | | | |
| Asthma5Y ~ 1 + LRTI<18m | | | | | Asthma5Y ~ 1 + LRTI Severity | | | | |
| LRTI<18m |  |  |  | | LRTI Severity | |  |  |  |
| *Yes* | 1.18 | 0.74 – 1.89 | 0.481 | | *Mild* | | 1.10 | 0.69 – 1.78 | 0.680 |
| - |  |  |  | | *Mod-Severe* | | 3.76 | 1.01 – 11.16 | **0.027** |
| Asthma5Y ~ 1 + Non-respiratory Antibiotics | | | | | Asthma5Y ~ 1 + Non-respiratory Antibiotics | | | | |
| Non-resp ATBX | 1.08 | 0.44 – 2.30 | 0.851 | | Non-resp ATBX | | 1.08 | 0.44 – 2.30 | 0.851 |
| Mediator model:  Non respiratory antibiotics (M) ~ LRTI <18m (X) | | | | | Mediator model:  Non- respiratory antibiotics (M) ~ LRTI severity (X) | | | | |
| ATBX ~ 1 + LRTI<18m | | | | | ATBX ~ 1 + LRTI Severity | | | | |
| LRTI<18m |  |  |  | | LRTI Severity | |  |  |  |
| *Yes* | 0.82 | 0.57 – 1.18 | 0.289 | | *Mild* | | 0.79 | 0.54 – 1.14 | 0.205 |
| - |  |  |  | | *Mod-Severe* | | 2.29 | 0.64 – 6.37 | 0.147 |
| Outcome model:  5-year Asthma (Y) ~ Non-respiratory antibiotics (M) + LRTI<18m (X) | | | | | Outcome model:  5-year Asthma (Y) ~ Non-respiratory antibiotics (M) + LRTI severity (X) | | | | |
| LRTI<18m |  |  |  | | LRTI Severity | |  |  |  |
| *Yes* | 1.18 | 0.74 – 1.89 | 0.476 | | *Mild* | | 1.11 | 0.69 – 1.78 | 0.677 |
| - |  |  |  | | *Mod-Severe* | | 3.75 | 1.01 – 11.14 | **0.027** |
| Non-resp ATBX | 1.09 | 0.44 – 2.32 | 0.831 | | Non-resp ATBX | | 1.04 | 0.42 – 2.23 | 0.917 |
